# Obesity-associated metabolic syndrome spontaneously induces infiltration of pro-inflammatory macrophage in synovium and promotes osteoarthritis

**DOI:** 10.1371/journal.pone.0183693

**Published:** 2017-08-31

**Authors:** Antonia RuJia Sun, Sunil K. Panchal, Thor Friis, Sunderajhan Sekar, Ross Crawford, Lindsay Brown, Yin Xiao, Indira Prasadam

**Affiliations:** 1 Institute of Health and Biomedical Innovation, School of Chemistry, Physics, Mechanical Engineering, Queensland University of Technology, Brisbane, Australia; 2 Institute for Agriculture and the Environment and School of Health and Wellbeing, University of Southern Queensland, Toowoomba, Queensland, Australia; 3 The Prince Charles Hospital, Orthopedic Department, Brisbane, Australia; SERGAS and IDIS, SPAIN

## Abstract

**Objectives:**

Epidemiological and experimental studies have established obesity to be an important risk factor for osteoarthritis (OA), however, the mechanisms underlying this link remains largely unknown. Here, we studied local inflammatory responses in metabolic-OA.

**Methods:**

Wistar rats were fed with control diet (CD) and high-carbohydrate, high-fat diet (HCHF) for period of 8 and 16 weeks. After euthanasia, the knees were examined to assess the articular cartilage changes and inflammation in synovial membrane. Further IHC was conducted to determine the macrophage-polarization status of the synovium. In addition, CD and HCHF synovial fluid was co-cultured with bone marrow-derived macrophages to assess the effect of synovial fluid inflammation on macrophage polarisation.

**Results:**

Our study showed that, obesity induced by a high-carbohydrate, high-fat (HCHF) diet is associated with spontaneous and local inflammation of the synovial membranes in rats even before the cartilage degradation. This was followed by increased synovitis and increased macrophage infiltration into the synovium and a predominant elevation of pro-inflammatory M1 macrophages. In addition, bone marrow derived macrophages, cultured with synovial fluid collected from the knees of obese rats exhibited a pro-inflammatory M1 macrophage phenotype.

**Conclusion:**

Our study demonstrate a strong association between obesity and a dynamic immune response locally within synovial tissues. Furthermore, we have also identified synovial resident macrophages to play a vital role in the inflammation caused by the HCHF diet. Therefore, future therapeutic strategies targeted at the synovial macrophage phenotype may be the key to break the link between obesity and OA.

## Introduction

Accumulating epidemiological and experimental evidence supports an association between obesity and a higher incidence of osteoarthritis (OA) [1–3]. The contribution of obesity to the development of OA is intuitive as an increased load on the joints; however, bio-mechanics fail to explain the occurrence of OA in non-weight bearing joints in obese subjects. Increased plasma concentrations of insulin and insulin-like growth factors (IGFs), sex hormones and adipokines released from adipose tissue may influence OA directly through increased joint degradation; however, there is no clear consensus regarding the specific role of these factors in OA [4–9]. Consequently, the precise molecular mechanisms by which obesity influences OA development continues to be an important and unanswered question in OA research.

Increasing rates of diet-induced obesity have been attributed to the metabolic osteoarthritis, and metabolic OA has been proposed as a new phenotype of OA that displays a unique OA characteristics [10–13]. In order to understand and investigate metabolic OA, various diet-induced obesity (DIO) models have been established and validated. In a high fat, high sucrose (HFS) DIO rat model, after a 12-week post-obesity induction, DIO rats demonstrated increased OA-like cartilage changes, and systemic and local synovial fluid inflammatory markers and accumulation of adiposity [13]. Another study showed that mice exposed to high fat (HF) diet showed elevated OA scores, hyperalgesia, adipocyte-related hormones, and pro-inflammatory cytokines in serum in proportion to body fat, and physical therapeutic approaches produced modest changes in knee histopathology [14, 15]. Additional study found that obesity and dietary fatty acid content regulate the development of OA [16]. These studies together provide strong evidence that obesity is strongly linked with OA development.

Chronic inflammation is increasingly appreciated as a major factor promoting insulin resistance and other metabolic disorders associated with diet-induced obesity in adipose tissue [17–19]. Excess caloric intake leads to low-grade inflammation that is characterised by the presence of infiltrating inflammatory cells, such as macrophages, in adipose tissue [20]. Recently, it has been shown that certain components of MetS can alter the inflammation in joint structures. For example, excessive lipid diffusion into the joints via systemic circulation and synovial fluid were linked to cartilage matrix protein oxidation and increased synovial permeability [21]. Increased cholesterol levels strongly elevate synovial activation and ectopic bone formation in early-stage collagenase-induced OA [22]. Glucose concentration is strongly associated with catabolic and anabolic metabolism of chondrocytes and synovium as it is an essential substrate for global joint [9]. Hamada and colleagues provide evidence that TNFα is elevated in the synovium of obese Type II diabetic OA patients, but not in non-diabetic obese OA patients [23]. Increased serum glucose concentration has detrimental effects on cartilages and these effects are induced by overexpression of matrix metalloproteinase, reactive oxygen species and glycan end products, which results cartilage matrix breakdown and cell senescence [24, 25]. These results together suggest that certain components of metabolic syndrome can activate the synovial alterations inflammation and cartilage changes.

Macrophages are heterogeneous and remarkably plastic, generally inhabiting two major sub-populations: those in a predominantly M1-polarised pro-inflammatory state and those in a predominantly M2-polarised anti-inflammatory state [26]. In synovial tissue within the joints, macrophages are, along with fibroblasts, resident cells that under normal circumstances remain quiescent. Clinically, the local synovial inflammation in OA patients is highly variable, with some patients presenting no inflammation and others with severe inflammation [27]. Although obesity leads to inflammation, the detailed cellular events underlying the inflammatory changes at the onset of obesity in the local joint environment are not well-established.

In this study, we demonstrate that diet-induced obesity promotes macrophage infiltration and also activates macrophages towards the pro-inflammatory M1 phenotype within the synovium. We also show that macrophages and chondrocytes stimulated with synovial fluid harvested from obese rats produce inflammatory and degradative changes. These observations provide evidence that traditional western diet-high carbohydrate high fat is a risk factor for OA-like pathological changes. The mechanisms responsible for these interactions are not fully understood, but may involve M1 polarized synovial macrophage in the inflamed synovium. This research will provide a new overview of the involvement of synovial macrophage in promoting inflammatory and destructive responses in diet-induced obesity related OA and might therefore by used as a therapeutic strategy for the development of disease-modifying anti-OA drugs.

## Methods

### Rats

The use of rats for this study was approved by the Animal Ethics Committees of the Queensland University of Technology and the University of Southern Queensland under the guidelines of National Health and Medical Research Council of Australia (AEC project approval code: 14REA010). 30 male Wistar rats (9–10 weeks old) weighing approximately 330-350g were purchased from Animal Resource Centre (Perth, WA, Australia). The rats were individually housed at the University of Southern Queensland Animal House in a temperature-controlled, 12-hour light/dark cycle environment with ad libitum access to water and experimental diets. The rats were divided into three groups of ten animals each. One group was fed on HCHF diet for 8 weeks, and two other groups were fed on CD or HCHF diets for 16 weeks. Physiological measurement and metabolic parameters were collected and analysed as described [28, 29]. The two experimental diets used in this study were a control diet (CD) and a high-carbohydrate, high-fat diet (HCHF). The composition of the diets used in this study were same as described in our previous study [28, 29].

### Tissue harvest and histologic analysis

After 8 or 16 weeks of dietary interventions, the rats were euthanized by intraperitoneal injection of Lethabarb^®^ (100mg/kg). The knee joints were harvested and fixed in 4% paraformaldehyde followed by decalcification in 10% ethylenediaminetetraacetic acid and was later embedded in paraffin. Five-micrometer-thick sagittal sections were cut using a rotary microtome (Leica). The rat knee sections were stained with fast green and Saf-O and then evaluated for cartilage damage and synovial inflammation by two independent assessors. The pathological changes of joint were assessed using the Mankin scoring system, as previously described [22, 30]. Synovial thickening was assessed using a 0–3 scoring system as previously described [22, 31](0 = no synovial thickening; 1 = lining of two cell layers; 2 = several extra cell layers; 3 = clear inflammation with cell infiltrate). The criterion used for the selection of the four target sites were, lateral femur (LF), lateral tibia (LT), medial femur (MF) and medial tibia (MT).

### Preparation of synovium homogenates and total RNA extraction

After the rats (n = 5) were euthanized, the synovium of knee joints were isolated and cut into pieces. The samples were snap frozen in liquid nitrogen and homogenized in 1ml QIAzol lysis reagent from Qiagen (Doncaster, VIC, Australia). Total RNA was extracted from homogenates by using RNeasy Lipid Tissue Kit (Qiagen, Doncaster, VIC, Australia) according to the manufacturer’s instruction. The RNA quantity and quality were assessed using a Nanodrop 1000 Spectrophotometer (Thermo Scientific, Scoresby, VIC, Australia). Reverse transcription was then followed.

### Immunofluorescence and immunohistochemistry (IHC)

For immunofluorescence, a standard two-step staining technique as described [21]. Sections were incubated with rabbit anti-rat CD68 antibody (Abcam, Melbourne, VIC, Australia; dilution 1:250), rabbit anti-rat iNOS antibody (Thermo Scientific, Scoresby, VIC, Australia; dilution 1:500) and a rabbit anti-rat Arg1 (Abcam, Melbourne, VIC, Australia; dilution 1:100). The sections were incubated with corresponding fluorescent secondary antibodies. Immunofluorescence was examined with a Leica SP5 confocal microscope.

Immunohistochemistry was performed using standard protocols [30]. The various antibodies and their concentration used in this experiment were similar to immunofluorescence with the substitution of secondary antibodies (Dako, North Sydney, NSW, Australia). Samples were stained with diaminobenzene and counterstained with haematoxylin. Images were captured using a Zeiss Axio vision light microscope. To conduct semi-quantitative data analysis, the positive cells from different fields of observation were counted and normalized to the cell number per 100 total cells in each group.

For each immunostaining, negative control either without primary antibody or with isotype-matched IgG instead of primary antibody was included. All sections were randomly coded and scored in a blinded way by two independent investigators.

### Collection of knee joint synovial fluid from rats

Immediately after euthanasia, The skin overlying the knee was excised from both control and obese rats, the knee joint cavity was entered by a 27G needle and 100 μL sterile saline which given into the joint was withdrawn and taken into a 1.5ml centrifuge tube. Synovial fluid (SF) aspirates with sterile saline from both knee joints was pooled. Synovial fluid samples were centrifuged at 700× g for 10 minutes according to published study [32] to remove cellular elements and then immediately frozen at -80°C until further use. The presence of cellular elements in synovial fluid were manually determined at a high magnification (40 ×) using synovial fluid smears.

### Rat bone marrow-derived macrophage (BMDM) cell culture

Rat BMDMs were isolated and cultured using published protocol [33]. The femur and tibia were obtained from CD diet-fed rats and bone marrow was collected by flushing the bones with serum-free DMEM supplemented with 1% penicillin-streptomycin using a 10-ml syringe and a 24-gauge needle. Bone marrow cells were collected by filtering through a 40μm cell strainer (BD Biosciences, North Ryde, NSW, Australia). Monocytes were stimulated with 20 ng/ml recombinant macrophage colony-stimulating factor (M-CSF; 100ng/ml, PeproTech, Mt Waverly, VIC, Australia), then seeded in cell culture flasks (T75 cm^2^ flasks). All non-adherent cells in the supernatant were removed on day 3 and the remaining adherent cells (regarded as macrophages) were maintained in culture for a further 7 days with media being replaced every 3 days.

### Macrophages (BMDMs) co-culture with synovial fluid

Rat BMDMs were transferred to 24-well plates (2×10^6^ cells/ml) and incubated at 37°C for 24 h. The SF from HCHF or CD rats were diluted 1:1 with serum-free DMEM containing 1% penicillin-streptomycin. After 24 h, the adherent cells were washed and stimulated in serum-free medium, with either (1) HCHF SF, (2) CD SF, or (3) no stimulation at 37°C for 24 hours. The cells were washed and the polarisation state of the macrophages was determined by qPCR.

### Micromass cultures of rat articular cartilage chondrocytes (ACCs)

High-density micromass droplets were prepared as described previously [34]. The SF from CD or HCHF animals was diluted 1:1 with serum-free medium and added to the chondrocyte micromass. Controls received serum-free medium. After 72 h, the cells were washed and the effects of co-culture on the chondrocytic phenotypes was assessed by qPCR at 7 days. All experiments were performed using freshly isolated P0 cells.

### Isolation of synovial macrophages from OA synovium

Synovium was isolated from five patients (68.6 ± 8.6 years; 5 female; BMI 39.68 ± 3.625) with advanced clinical OA, all of whom were undergoing total knee replacement at The Prince Charles and Holy Spirit Northside Private Hospital, Brisbane, QLD. Ethical approval was granted by the Queensland University of Technology and The Prince Charles Hospital Ethics Committee. OA synovium were isolated and digested as previously described [35–37]. To obtain synovial macrophages, synoviocytes were separated based on cell marker CD14 [38]. Synoviocytes were incubated with CD14 microbeads (Miltenyi Biotec, Macquarie Park, NSW, Australia) in the dark at 4°C. The CD14 magnetically labelled cells were isolated by magnetic activated cell sorting (MACS, MASC Separation columns LS; Miltenyi Biotec, Macquarie Park, NSW, Australia). The selected macrophages were cultured in DMEM supplemented with 10% FBS at 37°C for 24 hours.

### Macrophage differentiation and co-culture

To obtain M1/M2 differentiated macrophages, human CD14+ synovial macrophages were simulated as describe [39]. A modified macrophage-chondrocyte co-culture system was performed. To obtain M1/M2 differentiated macrophages, human CD14+ synovial macrophages were transferred to 6-well plates and cultured for another 24h. Cells were stimulated in serum-free medium with either (1) 100ng/ml LPS (Sigma-Aldrich, Castle Hill, NSW, Australia) plus 20ng/ml IFNγ (R&D System, Noble Park, VIC, Australia) for M1 differentiation, (2) 20ng/ml IL-4 (R&D System, Noble Park, VIC, Australia) for M2 differentiation, or (3) no stimulation (control) at 37°C for 48 hours The differentiated macrophages were washed and cultured for another 24h in serum-free DMEM medium without IFNγ/LPS or IL-4.

For co-culture experiments, chondrocyte cell line (C28/I2) was cultured at 1×10^6^ cells per ml in 6-well culture plates and incubated at 37°C. After overnight seeding in regular growth medium (DMEM supplemented with 10% FBS and 1% penicillin-streptomycin), the cells were washed with PBS for 3 times. The CM of in vitro differentiated macrophages was diluted 1:1 with serum-free medium and directly added to C28/I2 cells. Control group received serum-free medium only. After 72 h, the medium was replaced according to each group respectively for another 72h. At the end of the co-culture period, total RNA and conditioned media were harvested for further analyses.

### Glycosaminoglycan assay

The total amount of released GAG in the supernatant at day 3 and day 7 from primary cultured chondrocytes was quantified using GAG assay kit (Blyscan Assay Kit, Labtek, Brendale, QLD, Australia). The assay was performed following the manufacturer’s protocol.

### RNA extraction, real-time PCR and enzyme-linked immunosorbent assay (ELISA)

Total RNA was extracted using TRIzol reagent (Invitrogen, Mt Waverley, VIC, AUS) in accordance with the manufacturer’s instructions. cDNA was synthesised from 1 μg of the total RNA according to manufacturer’s protocol using an SensiFAST cDNA Synthesis Kit. Real-time quantitative PCR, using SYBR Green detection chemistry, was performed on the ABI 7500 Fast Real Time PCR system (Applied Biosystems, Thermo Scientific, Scoresby, VIC, Australia). The initial cycle was 50°C for 2 min and 95°C for 10 min followed by 40 cycles of 95°C for 15 s and 60°C for 1 min. Melt curve analyses of all real-time PCR products were performed and shown to produce a single DNA duplex. All samples were measured in triplicate and the mean value was considered for comparative analysis. Expression levels were calculated relative to the mean of experimental control, and beta-actin expression was used as the internal control. Quantitative measurements of all primers used in this study were determined using the (2^−ΔΔCt^) method, and GAPDH and β-actin expression were used as the internal controls, as described previously by our group [34, 40, 41]

Cytokine production, reflective of the pro-inflammatory and anti-inflammatory function of M1 and M2 macrophages, respectively, was assessed using ELISA. The ELISA kits were purchased from R&D systems (R&D System, In Vitro Technologies, Noble Park, VIC, Australia). The pro- and anti-inflammatory cytokine concentrations including IFN-γ, IL-1β and IL-10 in serum were also quantified using ELISA (R&D System, In Vitro Technologies, Noble Park, VIC, Australia). The various inflammatory cytokines used were (1) human: TNF-alpha and IL-10 and (2) rat: IL-6 and IL-10. The assays were performed following the manufacturer’s protocols for the specific cytokine.

### Statistical analyses

Statistical differences were tested using an unpaird Student’s *t* test for comparison of two parametric variables (i.e. body weight, metabolic parameters, gene expression), Mann-Whitney test for comparison of two non-parametric variables (i.e. histological scores). All analyses were performed using GraphPad Prism 7 and *P*-values < 0.05 were considered to be significant. All data are presented as mean ± SD.

## Results

### High carbohydrate and high fat (HCHF) feeding induces metabolic syndrome and obesity in the rats

**Fig 1:**
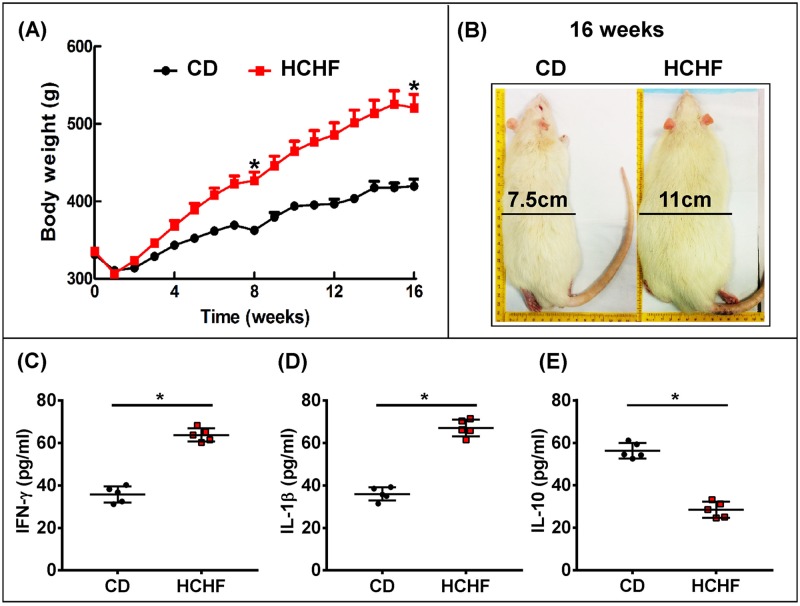
Metabolic effects of HCHF diet feeding. (A) Body weight of Wistar rats on CD or HCHF diet (n = 8). (B) Dorsal view of the rats showing the changes in the total abdominal length caused by the two diets after 16 weeks. ELISA analysis of pro-inflammatory (C) and (D) or anti-inflammatory (E) cytokines in serum (n = 6). Data were analyzed by two-tailed Student’s t test. All data are presented as mean ± SD. P < 0.05 (CD vs HCHF at two time point- week 8 and week 16) was considered to be significant. * = *p* <0.05.

We have previously reported that rats fed a HCHF diet develop symptoms characteristic of metabolic syndrome and cardiovascular changes, in particular central obesity, elevated blood pressure, impaired glucose tolerance, insulin resistance, non-alcoholic fatty liver disease and dyslipidaemia; this is therefore a relevant model of diet-induced metabolic syndrome in humans [28, 29]. In line with our previous studies, 9-week-old male Wistar rats fed a HCHF diet for 16 weeks had a significant increase in body weight and total abdominal fat (Fig 1A and Table 1) which led to an increased abdominal circumference gradually (Fig 1B and Table 1). Rats given CD diet for 16 weeks had higher food intake than the HCHF diet-fed rats, while the energy intake were lower in CD diet group due to low energy density of CD (Table 1). HCHF diet-fed rats had higher feed conversion efficiency than CD rats (Table 1). Total body fat mass was higher in HCHF diet-fed rats, with no difference in lean mass between the two groups (Table 1). The basal blood glucose concentrations and AUC were similar between HCHF and CD rats at 16 weeks. The variations were high for basal blood glucose concentrations in HCHF rats while variations for AUC were high for CD rats (Table 1). The plasma insulin concentration in 16 weeks HCHF diet-fed rats were increased than CD group (Table 1). These findings indicate that the rats were hyperinsulinaemic together with a decrease in insulin response which is the definition of insulin resistance. HCHF group also showed higher plasma activities of alanine transaminase, and alkaline phosphatase than CD rats (Table 1). Furthermore, plasma total cholesterol concentrations did not change, but plasma concentrations of triglycerides and NEFA were markedly higher in HCHF rats compared with CD rats (Table 1). Rats given a HCHF diet were also showed worsening cardiac function and increased systolic blood pressure when compared to CD rats, as described previously by our group [28]. We next compared the pro- and anti-inflammatory cytokine concentration in the serum of CD and HCHF rats. The HCHF diet-fed rats showed significant higher concentration of two pro-inflammatory markers IFN-γand IL-1β (Fig 1C and 1D), while the concentration of anti-inflammatory marker IL-10 was lower in HCHF compared to CD animals (Fig 1E), indicating a HCHF diet induced pro-inflammatory condition in animals by suppressing anti-inflammatory cytokine expression.

**Table 1:** Dietary intake and body composition in CD and HCHF rats.

Variables	CD (16 weeks)	HCHF (16 weeks)
Food intake (g/d)	40.2 ± 2.2	25.0 ± 2.3*
Water intake (mL/d)	26.1 ± 4.1	25.2 ± 3.5
Energy intake (kJ/d)	452 ± 24	543 ± 44*
Feed conversion efficiency (%)	19.3 ± 4.1	36.2 ± 6.8*
Total body fat mass (g)	79.5 ± 34.7	207.7 ± 81.6*
Total body lean mass (g)	305.0 ± 23.6	310.6 ± 46.9
Abdominal circumference (cm)	20.2 ± 0.7	22.4 ± 1.1*
Visceral adiposity index (%)	4.01 ± 0.88	10.07 ± 2.59*
Retroperitoneal fat (mg/mm)	155.2 ± 46.7	572.2 ± 219.1*
Epididymal fat (mg/mm)	98.0 ± 21.0	273.9 ± 69.5*
Omental fat (mg/mm)	88.7 ± 26.8	257.9 ± 73.9*
Basal blood glucose concentrations (mmol/L)	3.59 ± 0.30	3.86 ± 0.51*
Area under the curve (mmol/L·min)	687 ± 124	726 ± 73*
Liver (mg/mm)	203.2 ± 28.0	345.9 ± 33.6*
Alanine transaminase (U/L)	19.4 ± 5.4	40.9 ± 11.1*
Aspartate transaminase (U/L)	57.4 ± 13.4	66.4 ± 7.8
Alkaline phosphatase (U/L)	121.7 ± 24.8	248.7 ± 59.5*
Total cholesterol (mmol/L)	1.42 ± 0.36	1.59 ± 0.11
NEFA (mmol/L)	1.09 ± 0.37	4.84 ± 1.13*
Triglycerides (mmol/L)	0.36 ± 0.17	1.92 ± 0.67*
Insulin (μg/L)	2.35 ± 1.80	4.12 ± 1.09*
Leptin (μg/L)	3.21 ± 1.12	11.37 ± 3.80*
Cardiovascular function [28]		

CD- control diet-fed rats; HCHF-high-carbohydrate, high-fat diet-fed rats; NEFA-non-esterified fatty acids.

*P < 0.05 was considered to be significant.

All data are presented as mean ± SD. n = 8.

### HCHF diet induces OA-like pathological changes

**Fig 2:**
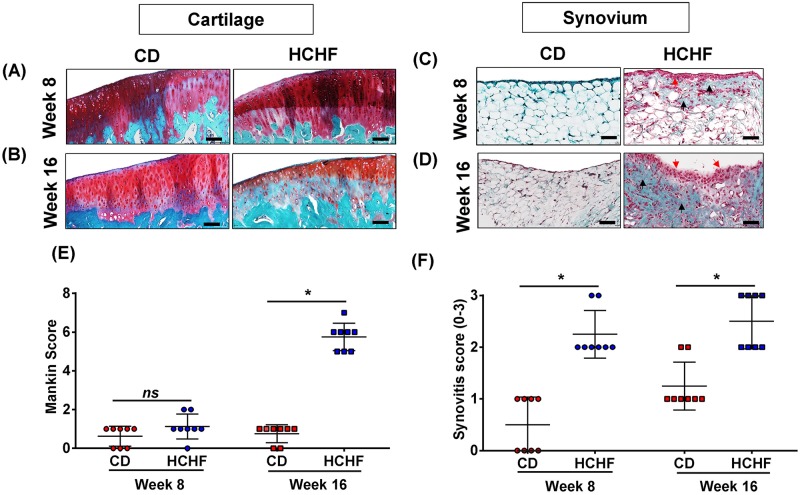
Time-dependent histopathologic changes in the joint of HCHF diet-fed rats (8 and 16 weeks). Histological evaluation of the knee joints. Tissues were stained with safranin-O and fast green (A-B) to estimate the proteoglycan loss among the two time points in HCHF and CD groups. Scale bar = 20 μM. Extent of articular cartilage degradation was graded using Mankin scoring system (E). Safranin-O and fast green staining shows the difference in thickness of the synovial membrane following, (C)8, and (D)16-week HCHF diet Histological scoring showed increased synovial thickening in 8 and 16-week-diet rats (F). Data were analyzed by Mann-Whitney test. All data are presented as mean ± SD. *P* < 0.05 (HCHF at week 8 vs HCHF at week 16) was considered to be significant. * = *p* <0.05. n = 8 per each group at each collection time point. Scale bar = 20 μM.

Effects of obesity and obesity-initiated metabolic syndrome on the knee joints assessed by Safranin-O staining showed no proteoglycan loss in the articular cartilage at 8 weeks and a marked reduction in the proteoglycan content at 16 weeks in HCHF group compared to the controls (Fig 2A and 2B). We performed a semi-quantitative histopathologic grading of the stained knee cartilage using modified Mankin scoring system [22, 30], which showed the HCHF group at 16 weeks consistently recording higher Mankin scores than the other groups (Fig 2E). Synovial thickening (red arrow) of lining cells of HCHF diet-fed rats were increased at 8 weeks, and then further increased at 16 weeks (Fig 2C and 2D). The fibrosis area (black arrow) was observed in the body of the intima and sub-intima at week 8, and noted to occupy the entire structure at 16 weeks (Fig 2C and 2D). Synovitis score, a measure for the amount of infiltrated cells and local proliferation, was markedly increased in HCHF rats when compared with CD rats (Fig 2F).

### HCHF diet results in synovial thickening throughout the joint

**Fig 3:**
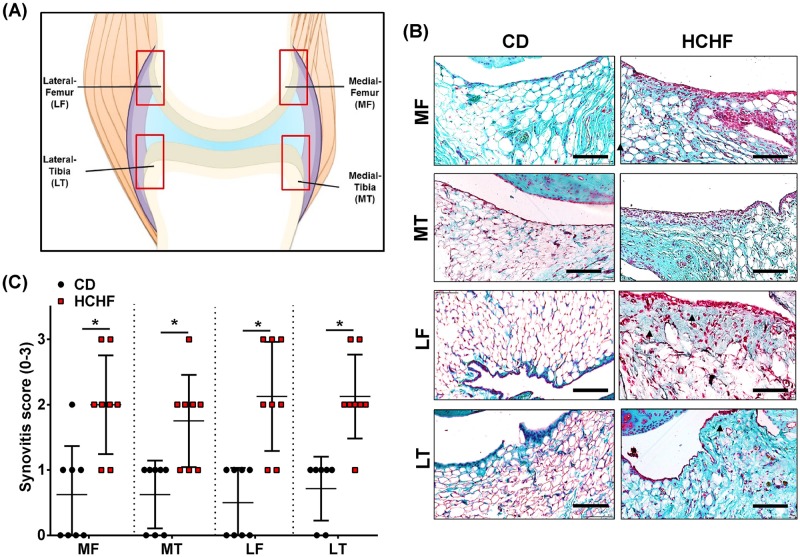
16 weeks of HCHF diet causes synovial inflammation. (A) Schematic diagram showing the Region of Interest (ROI). Safranin-O and fast green staining (B) shows the difference in thickness of the synovial membrane in the different regions of the knee joints of CD and HCHF diet rats. The synovitis score in the different regions of the knee joints of rats fed the two diets (C). Data were analyzed by Mann-Whitney test (CD MF vs HCHF MF; CD MT vs HCHF MT; CD LF vs HCHF LF; CD LT vs HCHF LF). All data are presented as mean ± SD, *P* < 0.05 was considered to be significant. * = *p* <0.05. n = 8 for each diet group at each ROI. Scale bar = 20 μM.

Next, we observed the synovial inflammation in four different sites of the synovial membrane (Fig 3A). These changes were characterised by increased thickness of synovial membrane layer and disorganised structure. Compared with the CD group, the knee joints of the HCHF group rats revealed marked synovial thickening. The synovial membrane was thickened by the presence of synoviocyte in the intima and sub-intima with a replacement of the connective tissue, from adipose to fibrous (black arrow) (Fig 3B). In contrast, the CD rats showed no signs of pathological changes of the synovium as indicated by typical palisading structure of the intimal lining layer and 2 to 3 layers of synoviocyte in the synovial intima and sub-intima with a predominance of adipose cells (Fig 3B). For each site of the synovial membrane, the synovial inflammation score was greater in the HCHF diet rats than in the respective control (Fig 3C). Together, these results indicate that the HCHF diet confers a systemic inflammatory phenotype resulting a global local inflammation.

### HCHF diet induces synovial polarisation

**Fig 4:**
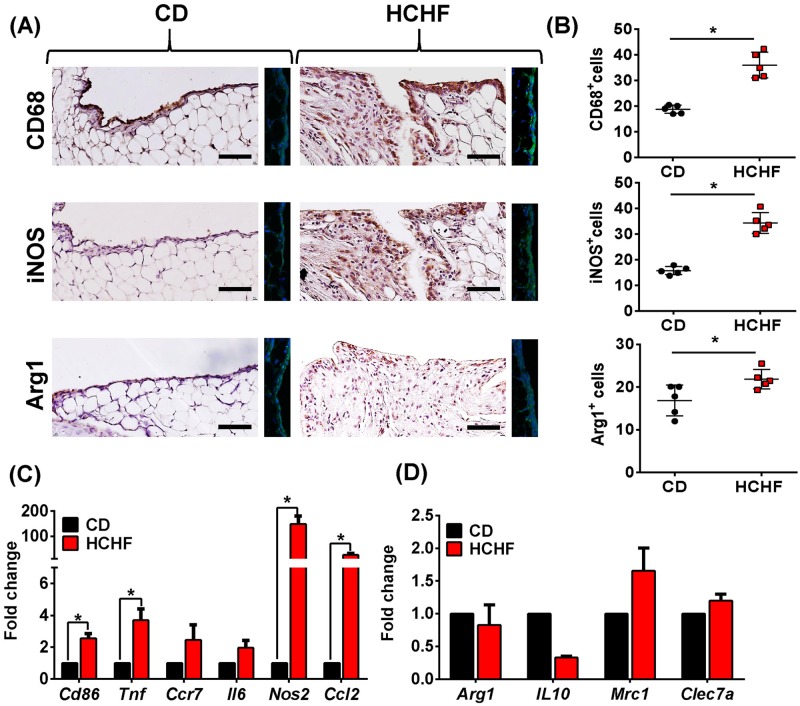
Macrophage-like cells increase in inflamed synovium of 16-week HCHF rats and are predominately iNOS+. (A) Representative immunohistochemical and immunofluorescence analyses of synovial tissues from CD or HCHF diet rats with anti-CD68, anti-iNOS or anti-Arg1. (B) Quantitative assessment of CD68+, iNOS+ or Arg1+ cells of inflamed synovium. Total positive cells per 100 cells were used as a standard measure to quantify. (C, D) qPCR analysis of pro-inflammatory M1-like (C) or anti-inflammatory M2-like (D) genes in synovium after diet stimulation. Data were analyzed by two-tailed Student’s *t* test. All data are presented as mean ± SD, *P* < 0.05 was considered to be significant. * = *p* <0.05. n = 5. Scale bar = 20 μM.

Our model was characterised by the evidence of inflammation and accumulation of cells in the synovium of HCHF diet-fed obese rats. To characterise the infiltrated cell population, immunohistochemistry was performed using a panel of phenotypic markers for macrophages. This analysis revealed increased macrophages in the synovia of the HCHF group compared with the controls (Fig 4). CD68-positive macrophages were increased in the synovial lining in the HCHF treatment group than in controls. Additional experiments were conducted to determine if increased CD68 expression in HCHF synovitis was related to preferential macrophage polarisation. The phenotype of the CD68+ macrophages resident in the synovium was assessed by the expression of the M1 marker iNOS and M2 marker Arg1. In the HCHF group, most cells in the synovial membrane were positive for the M1 macrophage marker and iNOS was expressed through the synovium, whereas the positive Arg1 marker was slightly increased in the intimal lining and sub-lining layers. Furthermore, the HCHF group had more intense iNOS fluorescent signalling compared with the controls, which was also the case with Arg1 (Fig 4A and 4B).

To further demonstrate that HCHF diet promote macrophage inflammation, we measured the mRNA expression of the different pro-inflammatory and anti-inflammatory genes by quantitative PCR (qPCR). HCHF diet resulted in higher inflammatory cytokine gene expression, as was observed in *Cd86*, *Tnf*, *Nos2* and *Ccl2* (Fig 4C), but did not significantly affect the expression of anti-inflammatory genes (Fig 4D), compared to CD diet-treated group.

### Synovial fluid of rats on HCHF diet alters macrophage polarisation and chondrocytes in vitro

**Fig 5:**
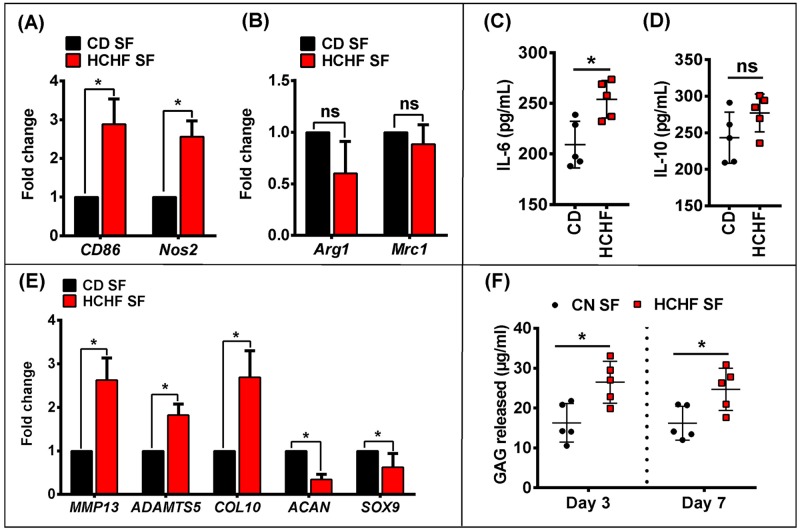
Synovial fluid of 16-week HCHF rats alters macrophage polarization and chondrocyte differentiation in vitro. Relative qPCR analysis of pro-inflammatory M1-like (A) or anti-inflammatory M2-like (B) genes in BMDMs after synovial fluid stimulation. ELISA analysis of pro-inflammatory (C) or anti-inflammatory (D) cytokines in conditioned medium. (E) Relative qPCR analysis of MMP13, ADAMTS5, COL10, ACAN and SOX9 in micromass cultured ACCs after 7 days of synovial fluid stimulation. (F) GAG release in supernatant of ACCs after synovial fluid stimulation at day 3 and day 7. Data were analyzed by two-tailed Student’s *t* test. All data are presented as mean ± SD, *P* < 0.05 was considered to be significant. * = *p* <0.05. n = 5 independent samples.

We then tested if synovial fluid from HCHF and CD altered normal rat bone marrow-derived macrophages (BMDMs) (Fig 5). Gene expression levels of *CD86* and *Nos2* were higher in these macrophages following treatment with HCHF synovial fluid indicating a polarisation towards an M1 phenotype (Fig 5A). There was a commensurate decrease in expression of *Arg1* and *Mrc1* in HCHF synovial fluid-treated macrophages compared with controls (Fig 5B). Further, IL-6 as an M1 marker cytokine increased in response to HCHF synovial fluid treatment (Fig 5C) whereas IL-10 expression as an M2 marker cytokine was not affected (Fig 5D).

We then tested whether obese rat’s synovial fluid affected chondrocytes directly and resulted in discernible *in vitro* phenotypic changes using a high-density micromass cell culture of rat articular chondrocytes stimulated in synovial fluid. The HCHF synovial fluid reduced gene expression of the chondrogenic markers *ACAN* and *SOX9* whereas expression of degradative and hypertrophic markers such as *MMP13*, *ADAMTS5* and *COL10* were increased compared to the CD synovial fluid (Fig 5E). Glucosaminoglycan (GAG) release into the medium as a measure of proteoglycan degradation was higher by day 3 in the HCHF group compared to CD and remained to be so after 7 days (Fig 5F).

### M1 polarised macrophages suppress chondrogenesis in articular chondrocytes

**Fig 6:**
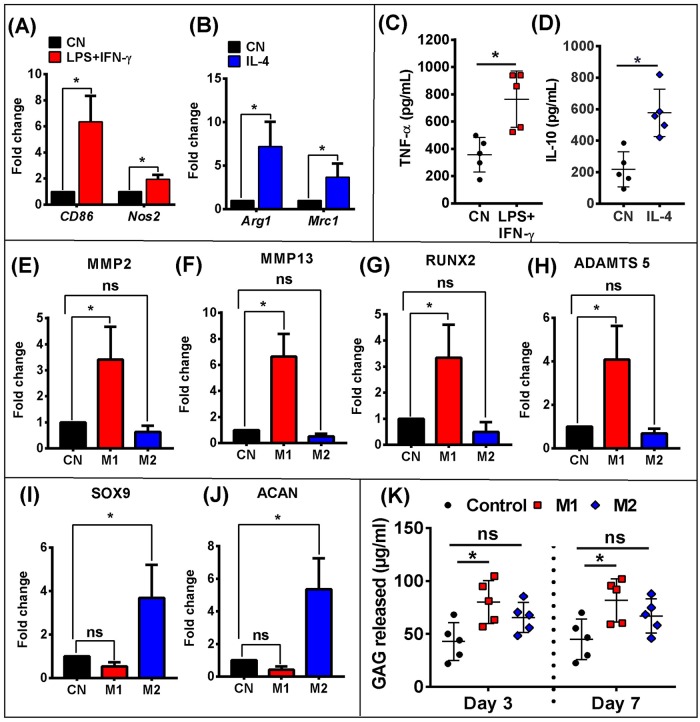
M1 macrophage negatively affects chondrogenic differentiation of chondrocytes. Relative qPCR analysis of pro-inflammatory M1-like (A) or anti-inflammatory M2-like (B) genes in CD14+ macrophages after cytokine treatment. ELISA analysis of pro-inflammatory (C) or anti-inflammatory (D) cytokines in conditioned medium. Relative qPCR analysis of MMP2 (E), MMP13 (F), RUNX2 (G), ADAMTS5 (H), SOX9 (I), and ACAN (J) mRNA levels in chondrocytes treated with M1 CM or M2 CM. (K) GAG release in supernatant of chondrocytes treated with 50% M1/M2 CM treatment for 3 and 7 days. Data were analyzed by two-tailed Student’s *t* test. Values represent the mean ± SD of experimental triplicates, *P* < 0.05 was considered to be significant. * = *p* <0.05. n = 5 independent samples.

The ability of polarised macrophages to decrease cartilage production was further tested in a series of experiments involving CD14^+^ synovial monocytes/macrophages. These cells were polarised towards an M1 phenotype by IFN-γ and LPS or alternatively towards an M2 phenotype by IL-4 (Fig 6). IFN-γ and LPS strongly upregulated the gene expression of M1 macrophage markers *CD86* and *Nos2* compared to controls (Fig 6A), whereas IL-4 increased expression of the M2 macrophage markers *Mrc1* and *Arg1* (Fig 6B). M1 macrophage polarisation resulted in higher concentrations of the pro-inflammatory cytokine TNF-α and reduced concentrations of IL-10 (Fig 6C and 6D).

The effects of polarised macrophages on chondrogenesis was assessed using the C28/I2 chondrocyte cell line cultured in a monolayer and treated with M1 and M2 conditioned media (CM). The gene expression of *MMP2* (Fig 6E), *MMP13* (Fig 6F), *RUNX2* (Fig 6G) and *ADAMTS5* (Fig 6H) was upregulated in response to M1 CM compared to controls. Conversely, expression of the chondrogenic markers, *SOX9* (Fig 6I) and *ACAN* (Fig 6J), was robustly upregulated by M2 CM but there was no change in the cartilage degradation markers. The results with M2 CM suggest that M2 macrophages prevent chondrocytes from de-differentiating into a hypertrophic phenotype. This hypothesis was further supported by the GAG release assay which showed increased GAG release resulting from M1 CM treatment (Fig 6K).

## Discussion

In this study, we subjected rats to a high-carbohydrate, high-fat diet to induce a metabolic state that mimics human obesity as part of metabolic syndrome. This diet consists of high amounts of simple sugars (such as fructose and sucrose) as well as long-chain saturated and trans fatty acids [28] which increased energy intake. The total caloric density of the high-carbohydrate, high-fat diet was considerably higher than standard corn starch chow. Although the overall food intake of obese rats was lower than that of the of the control rats, the overall energy intake was therefore higher for the high carbohydrate, high-fat diet-fed rats compared to the controls. The prolonged consumption of energy-dense diets in humans is strongly correlated with visceral obesity and the onset of metabolic syndrome, which suggests that our model is an appropriate model to explore the association between obesity and related complications including cartilage dysfunction [42–45]. With this model, we showed negative impact on the knee joints of the animals fed an energy-dense diet. Our results suggested that the HCHF diet cohort developed changes to cartilage homeostasis, exhibiting synovial inflammation resembling that in OA mediated by macrophages. These responses followed the dietary regimes and were not induced by surgical destabilisation of the joints, so indicating the hypothesis that synovial inflammation may be a key mediator and possible initiator of obesity-induced OA.

The accumulation of inflammatory T cells, B cells and macrophages are implicated in the tissue damage arising from high-fat diet-induced obesity by promoting adipose tissue inflammation and exacerbating insulin resistance [46–48]. There is growing evidence supporting the notion that synovial macrophage is the key effector cell for both inflammatory and destructive responses in OA [14, 33, 49]. The synovial macrophages are abundant in synovial intima and sub-intima, and are the dominant cell types present in the inflamed synovium occur in approximately 50% of OA patients [50–52]. Histological evaluation of inflamed OA synovium has shown that these cells express a high level of a general macrophage marker CD68, even in the early phase of the disease [51]. CD68, also known as Gp110 or macrosialin, is a 110-kDa glycoprotein highly expressed by monocytes and tissue macrophage and is considered as a standard pan marker [49]. Previous study has shown that *in vivo* depletion of synovial macrophage prior to induction of collagenase-injected experimental OA blocked cartilage destruction and osteophyte formation [53], which clearly indicates the involvement of macrophages in development of OA progression. Since inflammation plays an important role in the development and progression of OA in human and post-traumatic OA models, we aimed to test if obesity-related metabolic alteration in rodent model is associated with synovial macrophage activation and cartilage destruction. In the present study, rats subjected to the HCHF diet had a higher number of cells within the synovium of the knee joint that were immune-reactive for the macrophage marker CD68. It should be noted that the finding of increased OA-like changes was dependent on the presence of HCHF diet alone. This is consistent to a previous animal study demonstrating worsening knee OA in mice on a high fat diet [14]. A recent study, has shown that depletion of macrophage increases systemic and local inflammation and did not attenuate experimental OA in high-fat induced obesity in MaFia mice, however this study results were based only on short-term depletion, long-term depletion experiments are required to confirm the macrophage involvement in obesity induced OA [54]. Our finding suggest a local joint macrophage activation in the development of obesity-associated OA. However, we were unable to define the origin of these macrophages and whether or not they were differentiated from proliferating resident synovial cells or, alternatively, recruited from systemic blood cells. Since we found the alterations of synovium in the multiple sites of knee joints, the latter scenario is feasible; a cell-labelling study in mice showed that up to 5% of labelled blood monocytes were still expressing the Ki67 cell-proliferation marker two days after being transferred into recipient animals [55, 56]. As such, we noticed an increase of macrophage inflammation halfway through the 16- week-diet, when the cartilage still appeared normal. This indicated the immune response in the synovium preceded cartilage degeneration which became more apparent at the experimental endpoint.

Previous studies have shown that the macrophages are polarized and acquire a phenotype, ranging from pro-inflammatory (M1) to anti-inflammatory M2; of these polarized macrophages, classically activated M1 macrophage is the main contributor to produce pro-inflammatory cytokines which has been considered as a factor in mediating catabolic effects, while anti-inflammatory M2 macrophages exert anabolic effects [35, 57]. These findings led us to consider the spectrum of macrophage phenotypes in diet-induced obesity-driven synovitis. In order to distinguish and test the presence of M1 and M2 macrophages under obese conditions, we performed a single colour immunohistological staining by using defining markers, activate inducible nitric oxide synthase (iNOS) and type 1 Arginase (Arg-1), respectively. It is well established that iNOS and Arg-1 give rise to two mediators, the “killer” molecule nitric oxide (NO) and the “repair” molecule ornithine respectively that are involved in two opposite activities, pro-inflammatory (M1) verses anti-inflammatory (M2) function. Interestingly it was found that macrophages in synovium of obese rats preferentially expressed M1 marker as compared to those in the CD rats. On the other hand, M2-like macrophages were also slightly elevated in the inflamed synovium. The macrophage phenotypes were further confirmed by gene expression. It is well established in the literature that M1 polarized macrophages express pro-inflammatory markers *CD86*, *Tnf*, *Ccr7*, *Il6*, *Nos2* and *Ccl2*, in contrast, M2 activation leads to the expression of markers such as *Arg1*, *Il10*, *Mrc1* and *Clec7a* [58]. Along with analysis of immunohistochemistry, a mixed expression pattern of M1 and M2 macrophages was detected. However, HCHF diet did not significantly affect the expression of anti-inflammatory genes, compared to CD diet-treated group. One possible explanation is that in obesity model, adipose tissue secretes pro-inflammatory cytokines systemically that directly affect the local synovial macrophage alteration [59]. Furthermore, there is a strong interplay between iNOS and Arg-1, where ARG negatively regulates NOS activity by reducing the availability of l-Arg. On the other hand, Nω-hydroxy-l-arginine, an intermediate in the synthesis of NO, is a competitive inhibitor of ARG [60]. Even though M2 anti-inflammatory macrophages are only slightly increased in in inflamed synovium, we believe that the interaction of different subset of macrophages and other type of cells in synovium are important and required for catabolic and anabolic equilibrium.

Although the cytokine presence in the synovial fluid was extensively studied and a close relationship between cytokine expression and progression of OA was also showed in previous studies in experimental OA model and from patients with OA [61, 62], an interaction between synovial fluid microenvironment and synovial macrophages or chondrocytes in obesity-related OA remains unknown. In this study, we observed that synovial fluid of obese rats alters macrophage polarisation towards to a pro-inflammatory M1 phenotype in vitro, while the M2 polarization status was not sufficient. We observed that synovial fluid isolated from HCHF rats affected chondrocytes by upregulation of matrix degenerating genes. We also observed the significantly increased GAG release in chondrocytes exposed to HCHF synovial fluid. It might be possible that at the 16 week time point, the synovial fluid has already been conditioned by M1 macrophages, and observation that showed in chondrocyte co-culture system was potential positive feedback loop. It is known that the common characteristic linking obesity with OA is the “low-grade inflammatory state” [63], the importance of cytokines and adipokines in adipose tissue has been highlighted [64]. However, at this stage we were unable to identify specific stimuli included in the synovial fluid that was able to induce the pro-inflammatory profile in macrophages, and the pro-catabolic and pro-hypertrophic profile in chondrocytes. In previous studies it has been shown that the concentration of adipokines (leptin and resistin) was significantly higher in serum from obese individuals that were considered to be mediators that can act on synovial macrophages, infrapatellar fat pad inflammation and chondrocytes by regulating cartilage-degrading proteases, a disintegrin and metalloproteinase with thrombospondin motifs as well as pro-inflammatory cytokine and eicosanoids [65, 66]. Furthermore, the toll-like receptor 2 and 4 ligands such as S100-alamin proteins, fibronectin and low-molecular-weight hyaluronic acid has been found in synovial fluid, particularly early-stage OA that can induce catabolic responses in chondrocytes and inflammatory response in synovial macrophages [67–69]. Additionally, the pro-inflammatory cytokines like IL-1β, IL-6, IL-12 are associated with chondrocyte hypertrophy in OA, which induce release of GAG [70]. However, it was not certain whether these factors in synovial fluid alone stimulated the specific responses or whether one factor could influence the expression of another through systemic inflammatory response. In future, we will perform an overall profile of cytokines, chemokines, adipokines and other signalling proteins of the synovial fluid from the two group of animals to investigate the potential driver for macrophage and chondrocyte alternation.

Although we could not detect a clear M2 phenotype polarization after macrophage exposed to HCHF synovial fluid, the importance of the role of M2 macrophages during wound healing have been reported by previous studies such that the OA joint has been likened to a chronic wound [71–74]. For this reason, M1 and M2 CM prepared from human synovial monocytes/macrophages polarized to an M1 or M2 phenotype, were used as a model system to address the effect of both subsets on chondrocytes. We detected that M1 CM significantly increased degradative genes, where inhibition of these markers were not observed treatment with M2 CM. Although M2 CM treatment did not affect degradative genes, under non-inflammatory condition, the chondrogenic gene expression level were significantly increased by this stimulation. This was discrepant since it was demonstrated earlier that M1 polarized buffy coast isolated monocytes inhibited cartilage matrix genes and upregulated matrix degenerating genes *in vitro*, while M2 CM did not significantly affect any of these gene [57]. This published study focused on end-stage OA chondrocytes and have not taken into account the effect of polarized macrophages on the normal chondrocytes [57]. However, it is possible that the mechanisms associated to the effect of synovial fluid in macrophages and chondrocytes may not be the same to those activated by conditioned media in macrophages cells. A detailed metabolic profiling of synovial fluid and conditioned media will reveal the common mediator during this cross-talk. Our study has some limitations. Firstly, the cell phenotyping markers used in this study may also express in other cells. Although CD68 has been widely used to identify macrophages, its expression has been found in immature CD1a-postive dendritic cells [75]. iNOS has been found to be expressed in the peripheral blood lymphocytes and some dendritic cells showed higher expression of Arg-1 [76, 77]. Therefore, there is a possibility that some cells as identified by these three markers are not macrophages. Secondarily, the human CD14+ *in vitro* polarized macrophage phenotypes were characterized based on well-established markers. An extensive characterization of isolated CD14+ monocytes/macrophages may help to investigate which factor in OA synovial macrophages actually affected the cartilage under inflammatory condition. Moreover, in this study, we observed that HCHF synovial fluid, had remarkable effects on both synovial macrophages and chondrocytes, however, an overall profile of cytokines, chemokines and other signalling proteins of the synovial fluid from the two group of animals is required to clearly elucidate the association between the diet-induced obesity and OA. Additionally, we obtained joint synovial fluid sample by a standard lavage procedure [78]. Nonetheless, it is possible that different concentrations of synovial fluid analytes can be generated by the collection process alone, in addition to any changes due to obesity. Further alternative techniques [13, 79] may be required to obtain synovial fluid from small animal joints and fully clarify the effect of synovial fluid from HCHF rats to the negative impact of chondrocytes and alternation of macrophages. Additionally, similar as humans, Wistar rats demonstrate obesity prone and obesity resistance phenotypes after exposure to obesity-inducing diet [80, 81]. These two phenotypes has been used to evaluate the damage severity in different knee joints related to obesity. However, we do not find these two phenotypes in our studies, even though we used exclusively Wistar rats, as shown by the relatively narrow SD values for body weight and DEXA scans. In our recently published study [28], we found that different saturated fats can lead to differential weight distribution in Wistar rats. In future we will use these models to do a sub-analysis to determine the damage severity in response to rapid body fat and body mass across the outcomes.

## Conclusion

The key findings from this study are firstly, the importance of macrophage in the development of obesity-associated OA and secondly, the increase in M1 compared to M2 polarised cells in the obese rats compared to the lean cohort. It is, therefore, more than likely that the M1 macrophages are important in the development of obesity-associated OA. Further studies are needed to understand the initiation of macrophage polarisation and how macrophages interact with other factors involved in obesity, such as oxidative stress, complement activation, cell death and angiogenesis. Targeting these cells and their signalling pathways may be the key to discovering new interventions to break the obesity-OA link.
